# Fifteen Years of Gene Set Analysis for High-Throughput Genomic Data: A Review of Statistical Approaches and Future Challenges

**DOI:** 10.3390/e22040427

**Published:** 2020-04-10

**Authors:** Samarendra Das, Craig J. McClain, Shesh N. Rai

**Affiliations:** 1 Division of Statistical Genetics, ICAR-Indian Agricultural Statistics Research Institute, New Delhi 110012, India; samarendra.das@louisville.edu; 2 School of Interdisciplinary and Graduate Studies, University of Louisville, Louisville, KY 40292, USA; 3 Biostatistics and Bioinformatics Facility, JG Brown Cancer Center, University of Louisville, Louisville, KY 40202, USA; 4 Department of Medicine, University of Louisville, Louisville, KY 40202, USA; craig.mcclain@louisville.edu; 5 Hepatobiology and Toxicology Center, University of Louisville, Louisville, KY 40202, USA; 6 Alcohol Research Center, University of Louisville, Louisville, KY 40202, USA; 7 Department of Pharmacology and Toxicology, University of Louisville, Louisville, KY 40202, USA; 8 Robley Rex Louisville VAMC, Louisville, KY 40206, USA; 9 Department of Bioinformatics and Biostatistics, University of Louisville, Louisville, KY 40202, USA

**Keywords:** gene set analysis, microarrays, RNA-sequencing, genome wide association study, competitive, self-contained, sampling model, null hypothesis

## Abstract

Over the last decade, gene set analysis has become the first choice for gaining insights into underlying complex biology of diseases through gene expression and gene association studies. It also reduces the complexity of statistical analysis and enhances the explanatory power of the obtained results. Although gene set analysis approaches are extensively used in gene expression and genome wide association data analysis, the statistical structure and steps common to these approaches have not yet been comprehensively discussed, which limits their utility. In this article, we provide a comprehensive overview, statistical structure and steps of gene set analysis approaches used for microarrays, RNA-sequencing and genome wide association data analysis. Further, we also classify the gene set analysis approaches and tools by the type of genomic study, null hypothesis, sampling model and nature of the test statistic, etc. Rather than reviewing the gene set analysis approaches individually, we provide the generation-wise evolution of such approaches for microarrays, RNA-sequencing and genome wide association studies and discuss their relative merits and limitations. Here, we identify the key biological and statistical challenges in current gene set analysis, which will be addressed by statisticians and biologists collectively in order to develop the next generation of gene set analysis approaches. Further, this study will serve as a catalog and provide guidelines to genome researchers and experimental biologists for choosing the proper gene set analysis approach based on several factors.

## 1. Background

The advancement in genome sequencing technologies has led to the generation of tremendous volume of high-throughput and high-dimensional biological data [1]. Further, exploiting these data and drawing valid biological insights has posed a great challenge to researchers across the globe. For instance, in a gene expression (GE) study, the expression levels of several thousand(s) of genes are measured in a single experiment and further used for identifying the groups of genes which are relevant to the condition/trait under study [2,3,4]. Earlier, biologists considered this differential expression (DE) study as the end of their analysis [5]. However, such analysis is the starting point of a complex process of drawing valid biological insights into high-throughput genomic data [6]. Further, the DE analysis produces a list of associated genes ranked by the ascending or descending order of the magnitude of computed test statistic(s)/*p-values* (e.g., Z-score, fold change, t-test, etc.) [3,4,5]. This is a crucial step undertaken by the experimental biologists and genome researchers to select the informative genes as well as to obtain a global view of expression changes. Further, to put the long list of gene-level results into a broader biological context and to further reduce the complexity of analysis, secondary analytical approaches have been developed by grouping the long list of genes into smaller sets of related genes. One such approach is gene set analysis (GSA), and one of its popular forms is called as pathway analysis [7].

In the last decade, GSA has completely shifted the focus in GE and association data analysis from individual gene to gene set level [7,8,9,10,11]. Further, GSA has been extensively used in complex disease biology due to the polygenic nature of these disorders. GSA involves testing for association of sets of functionally related variants/genes, and can provide biological context for multiple genetic risk factors [12]. Recently, GSA was able to provide biological insights into mechanisms and possible treatment targets for complex diseases, including schizophrenia [13], bipolar disorder [14], Crohn’s disease [15], rheumatoid arthritis [16], breast cancer [17], and obesity [18]. Moreover, GSA has also been applied in plant biology to understand the abiotic stress response mechanisms in *Arabidopsis thaliana*, *Oryza sativa*, *Zea mays*, and *Gossypium raimondii* [9,10]. The GSA applications have led to novel biological hypotheses about the diseases/stress responses, and have suggested new avenues for molecular drug designing/crop breeding intervention [6,7,10,19,20,21,22].

Numerous statistical approaches and tools for GSA are now available for analysis of high-throughput genomic datasets. This includes GE data from microarrays and RNA-sequencing (RNA-seq) studies and single nucleotide polymorphism (SNP) data from genome wide association studies (GWAS). However, many researchers have tried to review the available GSA approaches in different times, but these are limited to only specific genomic studies. There is no comprehensive review of GSA approaches and tools meant for these broad spectra of datasets. Further, without sufficient understanding of the underlying statistical principles of GSA approaches, we may risk drawing erroneous biological interpretations and statistical conclusions. Moreover, there are minimal studies on grouping the available GSA approaches. Therefore, in this article, we aim to provide a comprehensive overview, statistical structure and steps concerning GSA approaches used for high-throughput genomic data analysis. Further, we classify the GSA approaches and tools based on the type of genomic study, null hypothesis, sampling model, nature of test statistic(s), etc. We also provide an overview of the evolution of GSA approaches in terms of different generations rather than reviewing them individually, along with their relative merits and demerits. Here, we address the key biological and statistical challenges in current GSA, which need to be addressed to develop the next generation of GSA approaches and tools.

## 2. Structure of Gene Set Analysis

**Figure 1:**
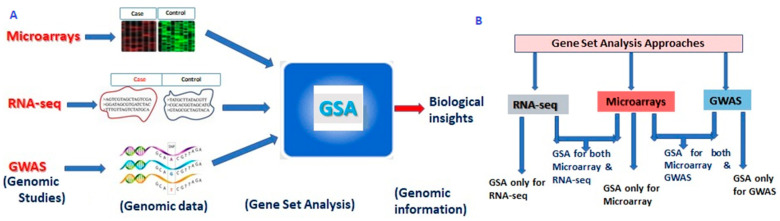
Outlines and classification of gene set analysis approaches. (**A**): Outlines of gene set analysis approaches; (**B**): Classification of gene set analysis approaches for high-throughput sequencing studies.

The term GSA refers to an analysis of set of genes and does not specifically mean modelling of the relations among genes in the gene set. Formally, GSA is defined as a secondary statistical approach used to test the involvement/enrichment of the gene sets with any biological process or pre-existing bio-knowledge base or quantitative trait. In other words, genes are aggregated to gene sets based on shared biological or functional properties or any pre-existing bio-knowledge base or quantitative trait [6]. These bio-knowledge bases include databases of molecular knowledge, i.e., molecular interactions, regulation, molecular product(s), and even phenotype associations or quantitative traits. A list of available bio-knowledge bases is given in Supplementary Table S1. In other words, GE and SNP datasets are used as input for GSA (in the presence of a annotation database) to provide valid biological insights into various complex diseases (Figure 1 and Figure S1) [7,23]. In fact, GSA has the potential to be used for all genomic data analysis, where the output is a long list of genes or transcripts. For instance, that long list of genes can even come from any upstream analysis including signatures of co-expressed genes from weighted gene co-expression network analysis [4].

### 2.1. Units of Gene Set Analysis

The functional unit of GSA is the gene set, which can be defined as any group of genes that share a particular property, i.e., involvement in a common biological process or any pre-existing bio-knowledge base [7,12]. Through GSA, a gene set that shares a common property is tested for its association with the trait or phenotype under study [24]. For this purpose, a wide range of GSA approaches and tools are available for high-throughput sequencing studies. These tools have differences in underlying statistical principles and practices, but there are similarities among the available tools in terms of statistical structure. For instance, GSA for GE studies has a two-tier structure [12,25]: (a) computation of gene level statistic(s); and (b) bi-variate statistical testing to compute the test statistic or *p-value* for the gene set. However, GSA for GWAS has a three-tier structure: (a) computation of SNP level statistics; (b) associating SNPs to genes and computing gene-level statistics from SNP statistics; and (c) computation of enrichment statistic or *p-value or* False Discovery Rate (FDR) for the gene set.

### 2.2. Hypotheses in Gene Set Analysis

The available statistical approaches for GSA greatly vary with respect to underlying statistical tests and hence depend on the formulation of the null hypothesis [6,11,23]. These null hypotheses can be grouped as self-contained and competitive [26]. In the usual set up of GE studies (or GWAS), genes (or SNPs) that are significantly associated with a trait/phenotype are identified and then evaluated, whether the significantly associated genes (or SNPs) tend to cluster in predefined gene sets or not. For instance, the self-contained null hypothesis can be framed as, *H*_0_: genes/SNPs in predefined gene sets are not associated with the underlying trait (phenotype) against alternate *H*_1_: genes/SNPs in predefined gene sets are associated with the trait (phenotype). The statistical approaches with a self-contained null hypothesis are called as self-contained approaches of GSA and they only consider the genes (SNPs) in the predefined gene sets. Statistical tests of GSA with a competitive null hypothesis are known as competitive GSA approaches, and the underlying null hypothesis can be expressed as, *H*_0_: genes/SNPs in predefined gene sets are associated with the underlying trait (phenotype) as much as are genes/SNPs outside the predefined gene set, against *H*_1_: genes/SNPs in predefined gene sets are more associated with the trait (phenotype) than genes outside predefined gene set. Here, the competitive GSA approaches consider genes (SNPs) from both the predefined gene set and the outside gene set [6,10]. The self-contained null hypothesis is invariably more restrictive than the competitive null hypothesis.

### 2.3. Sampling Models in Gene Set Analysis

The enrichment significance of a gene set is assessed through *p-value or* adjusted *p-value or* FDR after multiple testing correction (i.e., lower values indicate more enrichment and *vice-versa*) computed from a statistical test. Further, these statistical tests are commonly based on experimental designs having subjects/genes as units. On such statistical designs, different sampling procedures are rigorously used to obtain the distribution of the test statistic(s). Here, two types of sampling models are used in GSA: (i) subject sampling model; and (ii) gene sampling model.

#### 2.3.1. Subject Sampling Model

Classical statistical tests are based on an experimental design having microarray/RNA-seq samples as subjects, where each subject has the same set of (GE) measurements [6,10,24]. In the usual supervised setting, the sampling model consists of *M* independent realizations (for *M* subjects) of (*X*_1_, *y*_1_), (*X*_2_, *y*_2_), …, (*X*_s_, *y*_s_), …, (*X*_M_, *y_M_*), where, *X_s_* represents the *N*-dimensional vector (*N*: total number of genes) of the GE levels for *s-th* subject and *y_s_* is the corresponding class label (e.g., case: +1 vs. control: −1), *s* = 1, 2, …, *M*. Therefore, *M* expression levels of different subjects are assumed to be independently and identically distributed (iid), but expression levels of genes within the same subject may be correlated for a given condition. Usually, resampling procedures like bootstrap and permutation procedures are used on such models for gene [4,27] as well as gene set testing [6,28]. The statistical combination of subject sampling model and a self-contained null hypothesis provides a reliable platform for valid computation of *p-values* with easy interpretation and close relation(s) with single gene (or SNP) testing [29].

#### 2.3.2. Gene Sampling Model

In GSA, 2 × 2 tables are extensively used to statistically fit a Hypergeometric distribution [6,30]. The underlying model of a 2 × 2 table is a gene sampling model. Further, each cell of such a table is filled with a sample of genes, each of which is drawn at random from the gene space (i.e., set of genes in the data). Here, in this sampling model, each sampling unit (i.e., gene) can be subjected to two fixed set of indicator measurements, i.e., (*A*, *B*), where, (i) *A* (1 or 0) indicates whether the gene is a part of the predefined gene set or not and (ii) *B* (1 or 0) indicates whether that gene is in the list of differentially expressed genes or not [6,10]. Further, the gene space can be formalized into a population having *N* units (for *N* genes) and shown as: (*A*_1_, *B*_1_), (*A*_2_, *B*_2_), …, (*A_i_*, *B_i_*), …, (*A_N_*, *B_N_*). The competitive null hypothesis is popular and easy to formulate in a gene-sampling model setup [23]. Here, the gene sampling model may be considered as a mirror image of classical subject sampling model [27]. The gene sampling model considers the sampling units as iid, which assumes that genes are independent. Such assumptions are highly unrealistic, and the *p-values* computed using such models are statistically invalid for further interpretations. Hence, gene sampling models are quite complex and delicate as compared to a subject sampling model and need the utmost care while using.

## 3. GSA Approaches for High-Throughput Genomic Studies

The GSA approaches can be grouped based on different high-throughput genomic studies, as the underlying nature and distributions of the datasets are different (Supplementary Table S2). A classification of GSA approaches with respect to their application to genomic studies is shown in Figure 1. Initially, the GSA approaches were developed for microarrays (i.e., microarrays GSA) and subsequently extended to RNA-seq and GWAS data analysis (Figure 1). For instance, gene set enrichment analysis (GSEA) was originally developed for microarrays, and subsequent extensions of GSEA, i.e., SeqGSEA and GSEA-SNP were introduced to analyze RNA-seq and SNP datasets respectively.

### 3.1. Microarrays GSA

**Figure 2:**
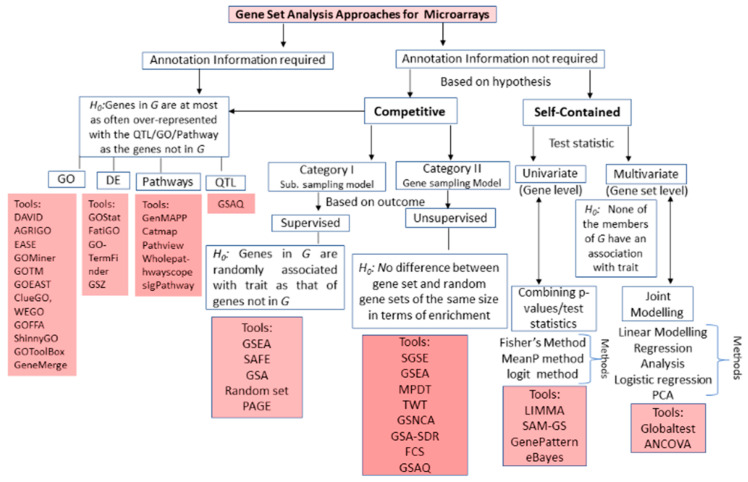
Classification of gene set analysis approaches and tools available for microarrays. Schematic representation of the breakup of GSA methods available for microarrays data analysis based on statistical tests (i.e., null hypothesis, test statistic(s)) and requirement of annotation databases. *G*: Gene set; * Tools require normalization of data prior to application.

Huge amounts of GE data from microarrays are available in public domain databases (Supplementary Table S3), which need to be analyzed for drawing valid biological insights into such datasets. Therefore, several GSA methodologies have been developed for this purpose. The classification of GSA microarrays is shown in Figure 2, which illustrates the evolution of GSA approaches over time in terms of the requirement of annotation information, sampling model, various null hypotheses under statistical tests. Moreover, the work on GSA started with the immediate need for functional analysis of microarray data based on gene ontology (GO) that gave rise to over representation analysis (ORA), which evaluates the statistical significance of gene sets in a particular pathway/functional category [21]. It is also referred to as a 2 × 2 table method [6], due to the fact that ORA approaches are mostly based on 2 × 2 tables and gene sampling models. The most commonly used statistical tests in ORA approaches/tools are hypergeometric, chi-square or binomial tests [20,31,32] (Supplementary Document S1). However, despite the extreme popularity and ease of execution, the ORA approaches also suffer from limitations, as listed in Table 1. The ORA form of analysis of gene sets can also be labelled as first generation of microarrays GSA.

**Figure 3:**
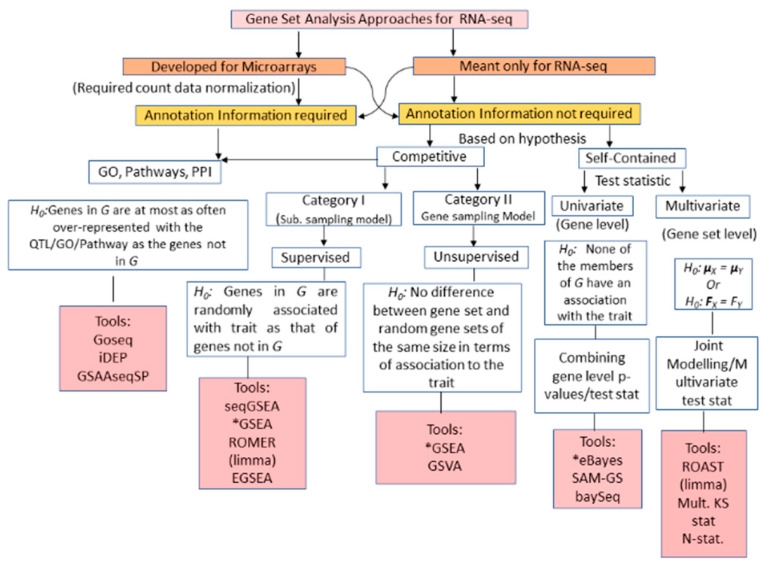
Classification of gene set analysis approaches and tools available for RNA-seq data analysis. Schematic representation of the breakup of GSA methods available for RNA-seq data analysis based on statistical tests (i.e., null hypothesis, test statistic(s)) and requirement of annotation databases. The first level of branching of the GSA methods based on their adaption from Microarrays practice to fit RNA-seq data as well as those specifically designed for RNA-seq. Subsequent branching depends on the different null hypotheses they test. *G*: Gene set. * Tools require normalization of data prior to application.

In most of the cases, the gene annotation information is either incomplete or totally unavailable; therefore, another class of GSA approach was developed. These approaches include the Enrichment Score (ES) form of GSA [33], starting with the landmark work on enrichment analysis of gene sets (i.e., GSEA) [8,24]. Subsequently, several other statistical approaches, algorithms and tools were developed for assessing the significance of gene sets in interpreting the high-throughput microarray data. The ES based GSA approaches greatly vary among themselves with respect to underlying statistical tests and sampling models. However, there are also commonalities among these ES based approaches in terms of execution, which is given in Supplementary Figures S1 and S2. The major steps for such approaches include initial computation of the gene-level statistic(s) using GE data under two contrasting conditions (Figures S1 and S2). For instance, correlation of expression measurements with phenotypes/traits [34], ANOVA [35], Q-statistic [26], signal-to-noise ratio [24], t-statistic [3], fold change [36], Z-score [37], etc., are implemented in contemporary ES based tools. There is a wider choice for gene-level statistic(s), ranging from parametric to non-parametric, for GSA. However, the selection of a gene-level statistic has a negligible effect on identification of significantly enriched gene sets [30]. When there are few biological replicates available, a regularized statistic may be preferred [30]. The second step is aggregation of gene-level statistic(s) for all genes in a gene set into a single gene-set level statistic (Figure 3). This includes the computation of gene-set level statistic using multivariate or univariate techniques (Figure 2). The former accounts for interdependencies among genes, while the latter disregards the same among genes distributed across the gene set. The currently available ES based GSA approaches/tools include Kolmogorov-Smirnov (KS) statistic, weighted KS statistic [24,33], sum, mean, or median of gene-level statistic [38], Wilcoxon rank sum [39], Max-mean statistic [8], etc. under univariate category. Moreover, multivariate category includes global test, ANCOVA, etc. for computing gene-set level statistic [26]. Interestingly, multivariate statistic(s) are expected to have higher statistical power, but univariate statistic(s) actually show more power at a higher level of significance (e.g., 0.1%) in real biological data, and equal power as the former at lower level of significance (e.g., 5%) [40].

The third step is computation of statistical significance (*p-value*) or adjusted *p-value* or FDR to assess the enrichment of gene sets (for gene-set level statistic) (Figure S1). This step requires the formulation, as well as testing of the null hypothesis against alternate one. Based on the null hypothesis, the ES-based GSA approaches can be broadly divided into: (i) competitive approaches and (ii) self-contained approaches (Figure 2). Moreover, the competitive approaches can be further subdivided into two categories based on the available outcome information of class: (i) supervised approaches and (ii) unsupervised approaches (Figure 2). Mostly, the supervised competitive approaches use the subject sampling model to randomly sample the class labels of each sample and compare the genes in the gene set with those of its complement. Here, it may be noted that the supervised term is used as the class labels are known and these approaches use these class labels for sampling purposes. However, unsupervised competitive approaches used the gene sampling model to compute the *p-value* through comparing genes in gene set with the genes outside gene set. But self-contained ES-based GSA approaches use the permutation procedure to compute the *p-values* by permuting the class labels for each sample and comparing the genes in the gene set with itself, while ignoring the genes outside gene set. Here, it is evident that competitive ES-based GSA approaches have more statistical power as compared to self-contained approaches [8]. This may be due to the fact that competitive approaches require information on both genes in the gene set as well as genes not in the gene set [6]. Furthermore, the ES form of analysis of gene sets may constitute the second generation of microarrays GSA (Table 1). The background methodologies for the various generations of GSA is given in Supplementary Document S1.

### 3.2. RNA-seq GSA

Recently, transcriptome deep sequencing i.e., RNA-Seq has surpassed microarrays by providing better quantification of GE for very high and low expressed genes (in terms of read counts), and higher levels of accuracy and reproducibility [11,78,79]. Hence, it is highly pertinent to adapt the existing microarrays GSA to RNA-seq data with the help of data transformation along with new approaches being developed (Figure 1B). The first approach of GSA for RNA-seq data (i.e., RNA-seq GSA), i.e., GOseq was suggested by Young et al. a decade ago [80]. It performs over-representation of GO categories enriched with a long list of highly expressed genes in RNA-Seq data. Further, an easy-to-use web application, integrated differential expression and pathway (iDEP) analysis was developed for in-depth analysis of RNA-seq data [81]. Detailed descriptions of the available RNA-seq GSA approaches, background methodologies, execution tools, and their features are listed in Table 2 and Supplementary Document S1. Moreover, the ORA-based RNA-seq GSA may be considered as the first generation of RNA-seq GSA.

To tackle the limitations of ORA approaches (Table 2), ES-based RNA-seq GSA approaches are developed, which constitute the second generation of RNA-seq GSA. Further, the major steps for RNA-seq GSA approaches are shown in Figures S1 and S3. Here, the read counts are given as input for computation of different test statistic(s) for GSA, which depend on the nature and distribution of the data. For instance, microarrays GSA (i.e., ES-based GSA) deal with continuous data expected to follow a Gaussian distribution (Supplementary Table S2) [78]. However, RNA-seq involves measurements that are non-negative counts ranging from zero to millions and are expected to follow negative binomial distribution (Supplementary Table S2) [11,79]. Therefore, microarrays GSA approaches may not be directly applicable to RNA-Seq data. Hence, some authors suggested normalization of the count data prior to the use of microarrays GSA [11]. For instance, VOOM-normalization is used for normalizing the read counts for sequence-depths, then microarrays GSA are applied on the normalized RNA-seq data [82]. The Goeman and Buhlmann formulation can be applied to classify the ES-based RNA-seq GSA approaches into either competitive or self-contained [6], based on the underlying null hypotheses (Figure 3). Further, a competitive GSA approach, i.e., gene set variation analysis (GSVA), was developed and demonstrated highly correlated results between microarrays and RNA-Seq sets for samples of lympho-blastoids cell lines [83]. This high correlation may be due to the fact that GSVA as a non-parametric approach does not depend on the distributional nature of data obtained from the studies. Fridley et al. proposed a GSA approach, i.e., gamma method (GM), with a soft truncation threshold to determine the significant gene set, while a generalized linear model is used to assess significance [84]. Subsequently, GSEA, the first ever competitive approach of RNA-seq GSA, was used for RNA-seq data analysis after normalization of the count data [84]. Thereafter, several modifications were made in GSEA by integrating both DE and differential splicing (DS) information in the analyses to develop SeqGSEA and has better performance over GSEA [28].

The self-contained GSA approaches can be divided into (a) univariate or gene-level; and (b) multivariate or gene set-level based on the distributional nature of the test statistic (Figure 3). The gene-level GSA approaches test a null hypothesis that the gene-set associated score does not differ between phenotypes/traits. Further, the univariate approaches are executed in two steps: (i) computation of gene level statistic(s) from the count data; and (ii) combining gene-level statistics to compute gene set level statistic or *p-value* or adjusted *p-value*. For the former case, the gene-level test statistic(s) of microarrays GSA were used in a recent study for RNA-seq GSA [84], which is quite straight forward and easy to implement. For the latter step, the gene-level statistic(s) can be combined into a single gene set statistic/*p-value* through Fisher’s method, Stoufer’s method, Meanp, logit method, etc. [10]. Moreover, the self-contained multivariate GSA approaches jointly model the genes to compute the gene set-level statistic(s) (Figure 3). These tests include multivariate generalization of the KS statistic [24,33], N-statistic [78], ROAST [82], etc. Further, the application of these tests requires the normalization of the RNA-seq data over varying sequencing depths [82]. Moreover, statistical significance is computed by comparing the observed statistics of gene sets with its null distribution, obtained by permuting the sample labels. Then, the enrichment significance of the gene set is assessed through the computed *p-value or* adjusted *p-value* or FDR after multiple testing correction.

### 3.3. GWAS GSA

**Figure 4:**
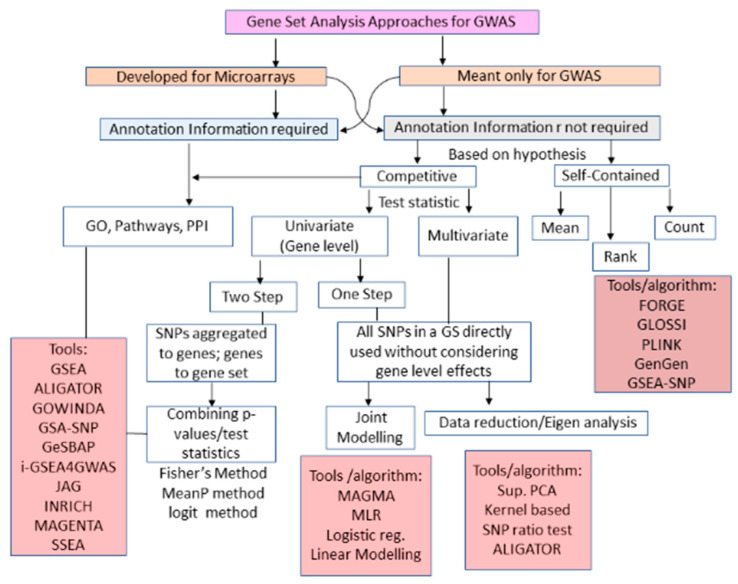
Classification of gene set analysis approaches and tools available for SNP data analysis. Schematic representation of the breakup of GSA methods available for SNP data analysis based on statistical tests and requirement of annotation databases. The first level of branching of the GSA methods based on their adaption from Microarrays to fit SNP data as well as those specifically designed for SNP data analysis. Subsequent branching depends on the different null hypotheses they test (i.e., null hypothesis, test statistic(s)). *G*: Gene set.

GWAS has been successfully applied to identify many novel loci for complex traits, which are quantitative (polygenic) in nature [17,18,19,20,21,22,41]. Therefore, to understand the underlying genetic architecture, GSA approaches have been used that place GWAS results in a broader biological context [91]. Initially, GSA methods for GWAS (i.e., GWAS GSA) were borrowed from microarrays [24,33] and subsequent new approaches were developed exclusively for GWAS (Figure 1). The classification of GWAS GSA approaches is shown in Figure 4. The first step for classification of GWAS GSA approaches can be their source of origin, including: (i) GSA microarrays adapted to GWAS; and (ii) those developed exclusively for GWAS (Figure 1 and Figure 4. Further, based on the requirement of annotation libraries, the GWAS GSA approaches can also be classified as: (a) GSA requiring pre-defined gene sets; or (b) GSA which does not require pre-defined gene sets. These approaches are based on the principle of over-representation of genes in those predefined gene sets obtained from different bio-knowledge bases (Table S1). Moreover, such ORA approaches constitute the first generation of GWAS GSA.

Due to the limitations of ORA-based GWAS GSA approaches, ES-based GWAS GSA approaches came into use, which we may call the second generation of GSA in GWAS. Their operational procedures and major analytical steps are given in Supplementary Figures S1 and S4. Further, the second generation of GWAS GSA starts with the enrichment analysis of gene sets for SNP data, i.e., GSEA-SNP [25,92] using weighted KS statistics [93]. Later approaches, based on other tests, *viz.* weighted-sum test [94], simple-sum test [95], collapsing test in combined multivariate and collapsing method [96] and sequence kernel association test [97], are used for computation of the gene-set enrichment score. Moreover, varieties of ES-based methods with similar ideas have been developed, such as the gene set based testing of polymorphism [98], GSA-SNP [92], SNP-ratio test [99], etc.

A class of GWAS GSA approaches have been developed by considering the topology of the gene sets/pathways, and this constitutes the third generation of GWAS GSA. This includes methods to parse the internal information of the pathway (e.g., signaling pathway impact analysis (SPIA) [74] and CliPPER [77]). Further, the second and third generation GWAS GSA methods focus on statistical results such as *p-values* or ES, as input rather than original data. Thus, the fourth generation of GWAS GSA approaches are developed by providing original data as input. Further, the underlying principle of these approaches is testing of the multivariate distribution of the multi-loci data or extracting the principal components from the original data. This includes linear combination test [100], supervised principal component analysis (SPCA) [100], Smoothed functional PCA [101], etc. Other model-based methods include LRpath [102], a logistic regression-based method, and MAGMA [103], linear model based method. Recently, the Generalized Berk-Jones (GBJ) statistic, a permutation-free parametric framework, was used for GSA [103], and this incorporates information from multiple signals in the same gene. The descriptions of the available GWAS GSA approaches, tools, their background methodologies pertaining to various generations are listed in Table 3 and Supplementary Document S1.

The formulations based on underlying statistical tests [6] can also be used for classifying GSA GWAS, i.e., self-contained and competitive approaches (Figure 4). Self-contained GWAS GSA considers only the SNPs in the gene set and tests the null hypothesis that none of those SNPs are associated with the phenotype. Competitive GSA considers all SNPs in the data and tests the null hypothesis that the genes in the gene set are no more strongly associated with the phenotype than other genes [128]. Further, the competitive GWAS GSA approaches can be divided into: (i) two-step approach(s), in which SNPs (in each gene) are first used to evaluate association with the gene, then gene-level statistic(s) are aggregated to gene-set level enrichment value to test its association with the phenotype; and (ii) a one-step approach, in which all SNPs in a gene set are simultaneously considered in the analysis without consideration of gene-level effects (e.g., MAGMA) (Figure 4). For the former categories the univariate statistical approaches are used, while multivariate techniques such as joint modelling are used for latter. Moreover, the self-contained GWAS GSA approaches can also be grouped based on the type of gene-set test statistic used for testing (Figure 4). This can be broadly subdivided into three classes: (i) mean-based, (i.e., mean or sum of the gene-association scores); (ii) count-based, (i.e., classifying genes as ‘significant’ or ‘not significant’ by applying a threshold to the gene-association scores and using the number of ‘significant’ genes in the gene set as a test statistic); and; (iii) rank-based, first ranking the genes according to their gene-association score and computing overrepresentation of the gene-set genes at the top of that ranking.

## 4. Limitations and Future Challenges of GSA

Here, we report the existing limitations as well as the key challenges observed in the available GSA approaches that should be kept in mind while using them. These existing limitations and challenges can be divided into two broad categories: (i) biological annotation challenges and (ii) methodological challenges.

### 4.1. Biological Annotation Challenges

The classification of GSA approaches for high-throughput genomic studies (Figure 2, Figure 3 and Figure 4) shows that GSA approaches require annotation information for analyzing gene sets. It is expected that the next generation GSA will require improvement of the existing annotations as well as new high-throughput annotation information [30,58]. Therefore, it is important to create accurate, high resolution bio-knowledge bases with specific emphasis on cell dynamics and condition, along with tissue information to annotate genes studied in an experiment. These knowledge bases will allow us to model the inherent organism’s response to any extraneous condition as a dynamic system and will help in predicting the system’s behavior at different times as well as in relation to various factors (e.g., mutation, disease, environmental conditions, etc.).

*Limited annotation information*: The contemporary GSA approaches mostly use GO and pathways information for analyzing gene sets [9,20,32,41,43,44,80,104,105], but there is enough other annotation information available or will soon be available in public domain databases that can be effectively used for GSA to gain biological insights into the etiology of complex diseases in humans as well as other organisms. A list of alternate annotation information along with possible hypotheses are listed in Supplementary Table S4. For instance, Das et al. used the quantitative trait loci (QTL) data as annotation information to develop a GSA approach to analyze the gene sets obtained from microarrays [10]. This approach has immense use for performing trait/QTL enrichment analysis of gene sets and further, QTL enriched gene sets can be used for molecular breeding programs for biotic/abiotic stress engineering in plants. Moreover, this annotation information can also be used in the future for developing new generation GSA approaches for analysis of RNA-seq and GWAS data. Such advances in GSA will open new avenues to understand the molecular complexity behind complex diseases in humans and other organisms including crop plants.

*Low resolution knowledge bases*: Recent advancement in genomics and proteomics leads to a paradigm shift in data generation, with unprecedented high resolution. At the same time, there is a demand for high resolution annotation bio-knowledge bases to perform GSA. For instance, during the early period of GE genomics, microarrays were the key experiment to obtain a global view of GE in the human genome. To perform GSA, GO [129] and KEGG [130] annotation bases were developed in parallel and implemented in several web tools. Further, such databases specify which genes (in terms of probe id/Enetrez id) are active in each GO category/pathway/any predefined gene sets. However, microarray technology has been replaced with RNA-seq and single cell RNA-seq (scRNA-seq) technologies. Hence, the current annotation databases need to be updated with respect to these high-resolution techniques. It is essential that they also begin specifying other information, such as transcripts (or scRNA-seq transcript) and SNPs that are active in each predefined pathway, GO category, etc.

*Missing or incomplete annotation*: Although enormous annotation bases are available in the public domain, some annotations are either missing or incomplete for certain genes. For instance, the current release of GO contained entries for 19,649 human genes annotated with at least one GO term. Many of these genes are hypothetical, predicted or pseudogenes. For example, the number of protein-coding genes in the human genome is estimated to be 20,000–25,000 [52], which shows that annotation information of hundred(s) of genes is still missing, and this may have a crucial role in various diseases. In addition to the missing annotations, most of the current databases have lower resolution (i.e., lesser information on transcript and SNP) [30,131], which leads to biased results from GSA. Further, current knowledge bases are built by curating experiments performed in different cell types at different time points under different conditions/locations. However, these details are typically not available in these knowledge bases. Thus, these databases need to be updated for future dynamic or cell specific GSA.

### 4.2. Methodological Challenges

*Lack of benchmark/gold standard*: In simulation, it is expected that multivariate approaches outperform the univariate counterparts, as the former considers inter-variable correlations. However, in biology, it is observed that univariate statistic(s) are equal to or better than multivariate statistic(s) [40]. This observation raises several questions about the performance assessment of GSA approaches using simulated datasets as a benchmark. It is likely that biology is more complicated than simulated scenarios and is influenced by factors such as the absence of exclusive division into classes, presence of outliers, experimental or technical hidden factors, environmental influence(s), random errors, etc. Therefore, one way to handle such a situation is to use benchmark/gold standard datasets with a valid biological basis. For instance, Ballard et al. (2010) compared two GSA methods based on their applications to three Crohn’s disease benchmark GWAS datasets with well-known biological basis [12,15,23]. Further, a combination of both benchmark biological datasets with statistically strong criteria can provide a suitable platform for comparative performance analysis of GSA approaches.

*Criteria for comparing GSA approaches*: When the performance of a GSA approach is assessed, it is expected to have certain proportions of false positives from the test. The ES-based GSA approaches compare the observed ES statistic with its null distribution as generated by random sampling/permuting the sample labels/disease outcomes or permuting genes/genotypes information [7,103]. Usually, through permutation, *p-values* are computed for assessing the enrichment significance of gene sets [6,26]. Then, −log10(*p-value*) and power of the statistical tests are used to assess the performance of GSA approaches [10]. However, alternate measures may also be used for comparative performance analysis of GSA approaches. In one such measure, the above computed *p-values* may be used to plot the histogram for the null gene sets, and that is expected to follow a uniform distribution. This phenomenon may be used to compute type-I error rates for GSA approaches, which can then be used as an efficient criterion for performance analysis of GSA approaches along with statistical power and FDR. In other words, GSA approaches with lower type-I error rates will be considered as better and *vice-versa*. These criteria can be computed on benchmark/gold standard datasets, which will provide a suitable platform to compare GSA approaches.

*Improvement in terms of statistical power*: In ORA-based GSA approaches, the test statistic(s) are computed by treating each gene equally. But in biology, some genes contribute more toward the disease/trait development. Treating all genes as equal in computing the test statistic reduces the statistical power of the GSA approach. Hence, one powerful strategy may be to consider the DE scores of genes [24,33,132,133] or ranks of the genes in a gene list while constructing the test statistic(s). This mechanism will attribute more statistical power to GSA approaches as compared to the existing ones. This approach needs to be well studied on benchmark data in future to assess its rigor and reproducibility. Further, other *a priori* biological information, *viz.* eQTL, network topology, co-expression scores, etc., can be used as auxiliary information in GSA approaches to improve their performance.

*Selection of null hypotheses:* The competitive GSA approaches use a gene sampling model to compute the *p-values* for gene sets [6,26]. In gene sampling model, it is assumed that genes are iid, which is highly unrealistic from a biological standpoint. Hence, the test statistic computed based on such assumptions from the gene sampling model leads to biased and misleading results. Therefore, methods, such as GSEA [24,33] and SAFE [39] use a hybrid concept, i.e., compute their test statistic(s) based on a gene-sampling model but calculate their *p-values* using the subject sampling model. The discrepancy between these two models makes the statistical properties of the test unclear and its interpretation very difficult. These problems are unavoidable, as the definition of the competitive null hypothesis is intimately tied to the gene-sampling model, whereas valid *p-values* are easily available for subject sampling only. This type of problem may provide impetus to future research in GSA.

*Inability to model and analyze a dynamic response*: It is well known that biological systems are dynamic. There has been a long debate about the feasibility of using static models to model the inherent dynamics of biological systems. However, in GSA, only static approaches (linear, gamma, generalized linear and regression models) [80,98,99] have been used so far. This raises a serious concern for the use of GSA approach in assessing living systems. The lack of methods that analyze gene sets as a dynamic system is partly due to the limitations of current molecular measurement technologies. These technologies can only quantify a snapshot of a biological system because they are unable to: (i) determine the protein states in a high-throughput fashion, or are severely restricted in this regard; and (ii) detect signals that propagate without affecting GE. Therefore, we encourage researchers in the future to use dynamic models such as time-series models, auto-regressive models, dynamic Bayesian models, etc. for GSA from time-dependent GE or association data.

*Redundancy among genes in gene sets*: In GE data analysis, redundancy among genes (i.e., genes may not be related to a case/disease but ranked in the top due to high correlation with other top ranked genes) is a serious issue [27]. During the process of ranked gene list preparation, redundant genes may be included and further, do not give valid *p-values* for the gene set testing, as genes in gene lists are correlated. In other words, *p-values* may easily be falsely significant when the genes in the gene set are correlated, even when none of the genes is truly significant. One strategy may be to use such a GE data analysis approach, (i.e., MRMR, Boot-MRMR [27]) which minimizes the redundancy among genes during the gene ranked list preparation. Other approaches may include avoiding the use of gene-sampling models in gene set testing for *p-value* computation. For this purpose, Goeman and Buhlman developed a subject-sampling 2 × 2 table method alternate to the gene sampling model to compute valid *p-values* for gene sets [6].

*Develop threshold-free approach(s):* ORA based GSA approaches are mostly threshold dependent [25]. Further, other GSA methods like mGSZ (based on Gene Set Z-scoring function) requires a threshold value for DE score to divide the ranked gene list into member genes and non-member genes (i.e., two gene groups) [132]. Gene set testing (e.g., Z-test) is then performed on these gene groups [15,24,33,132]. The determination of an optimal threshold is often a cumbersome task. Therefore, the obtained analytical results from such approach are unstable and irreproducible [24,25,93]. Hence, researchers use a set of threshold values to compute enrichment significance of gene sets and then select the threshold that gives the most significant results [6,134]. This approach seems inelegant. A more comprehensive and computationally intensive approach for choosing a threshold will be a reasonable compromise among power, type I error and reproducibility of results, using a cross validation technique. Another strategy may be development of threshold-free GSA approaches to improve the stability of results.

*Proper permutation procedure*: Current GSA approaches mostly use permutation procedures that compute *p-values* by comparing the observed test statistic with its null distribution generated from the permuted datasets [6,8,73,134]. It is expected to reflect chance-based confounding effects, including biases introduced by the gene set. However, the permutation procedures (if not designed properly) can produce misleading results and introduce bias in the resulting inference. For instance, permutation of SNPs, which is often used in *p-value* based approaches, may disrupt the linkage disequilibrium pattern and may not generate the correct null distribution. For gene-based approaches, permutation of sample labels may not generate the correct null distribution, as the samples are generated from tissues of same or related individuals [23,135]. Moreover, when the SNPs or genes or phenotypes are being permuted, the sampling units are assumed to be iid, which may not be the case; SNPs may be correlated due to linkage disequilibrium or gene-gene interactions. Therefore, proper care should be taken before choosing the permutation procedure for computing the *p-values* for gene sets.

*GSA approach(s) for alternate annotations*: The existing ORA-based GSA approaches have mostly focused on whether the selected gene sets are over-represented by known pathways or GO terms [9,20,32,41,43,44,80,104,105]. However, in plant and complex disease biology, such approaches may not able to establish any formal relation between the underlying genotypes and the trait/phenotype, as most of the traits are quantitative in nature and controlled by polygenes [10,12,13,14]. For this purpose, a statistical approach and R package of GSA with QTL has recently been developed [10], which is useful for obtaining QTL-enriched gene sets. Moreover, *like* QTL, there is a lot of genomic annotation information (Supplementary Table S4) available in public domain databases which can be used to develop new and innovative GSA approaches and tools.

*Stability of gene set testing results*: The statistical power and FDR are used for performance analysis of GSA approaches [7,8,11,78]. It is well known that different samples (on which the test is based) would give different results due to sampling errors. One way to deal with such a problem is to draw different sub-samples from a relative homogenous population, and the approach with small variance and uniform results over sub-samples can be termed as stable approach [16]. This principle can be applied to GSA, i.e., first, sub-samples can be taken from all samples, and then GSA can be applied on each sub-sample to compute the *p-value* for the gene sets. Finally, one can evaluate the stability of the approach by comparing a change in ranks over different sub-samples. The approach with the least change in ranks can be termed as the stable approach and can be easily implemented in simulation analysis. In biology, several factors may be responsible for causing instabilities to the results; these include, gene-gene correlations, genetic heterogeneity, and patient-to-patient variability. To address this problem, several researchers have hypothesized that testing gene sets rather than individual gene/marker will be more stable across different samples [8,136,137]. More relevant and specialized studies and methodologies are needed to validate such claims.

## 5. Discussion

In the last 15 years since its inception, GSA has become an extremely popular approach for secondary analysis of genome wide expression as well as association data. It has been successfully used to gain biological insights into the etiology of various complex diseases in humans as well as model organisms, including mammals, and other cellular organisms [9,10,13,14,138]. GSA has immense benefits in terms of biological interpretation of results, as well as numerous computational advantages over single gene studies [57]. It also enhances biologically meaningful interpretation of results and reproducibility of important gene lists yielded by independent studies, etc. [7,8,9,10,11]. In other words, the cumulative effects of the genetic variants (SNPs) or genes distributed in a gene set is considered in a single analysis and has more statistical power as compared to the univariate counterparts [8]. Despite of their usefulness, there are limited number of studies found in the literature, which consider the wider gamut of high throughput genomic studies from the GSA perspective. Hence, we have summarized the commonalities of GSA approaches used in three key genomic studies in terms of their execution, underlying null hypotheses, nature of test statistic, sampling models, etc. Further, the structure and key analytical steps common to most of the GSA approaches are discussed in this study.

Over the past few years, a diverse set of methods for performing GSA has been proposed for microarrays, RNA-seq and GWAS data analysis and the increased application of these methods has exposed several factors that affect the interpretations of GSA results. These factors include the null hypothesis being tested, the underlying sampling/permutation procedure, and the nature and distribution of test statistic(s). All of these factors play a significant role for choosing proper GSA for the data analysis. Researchers have also identified a variety of circumstances that can lead to faulty findings; hence, proper care is suggested to avoid misleading results. Several individual studies have been conducted over time to summarize GSA approaches for each type of genomic study [5,6,7,8,9,10,11,12,13,14,15,16,17,18,19,20,21,22,23,24,25,26,27,28,29,30,31,32,33,34,35,36,37,38,39,40,41,42,43,44,45,46,47,48,49,50,51,52,53,54,55,56,57,58,59,60,61,62,63,64,65,66,67,68,69,70,71,72,73,74,75,76,77,78,79,80,81,82,83,84,85,86,87,88,89,90,91,92,93,94,95,96,97,98,99,100,101,102,103,104,105,106,107,108,109,110,111,112,113,114,115,116,117,118,119,120,121,122,123]. Here, we summarize a comprehensive review of GSA approaches in terms of statistical structure, execution and classification for three different high-throughput genomic studies. Several approaches and tools have evolved over time, individually for each type of genomic study. Thus, instead of individually reviewing them, we present the classification of GSA approaches for microarrays, RNA-seq and GWAS into different generations along with underlying statistical methodologies/tests and special features. Many earlier reviews of GSA are data independent studies [6,11,23], but our study is data dependent and comprehensive.

This study will serve as a catalogue and provide guidelines to genome researchers and experimental biologists for choosing the proper GSA based on several factors. In this study, we reported several challenges which need to be addressed by statisticians and biologists collectively to develop the next generation of GSA approaches. These new approaches will be able to analyze high-throughput genomic data more efficiently in order to better understand the biological systems and to increase the specificity, sensitivity, utility, and relevance of GSA.

## Figures and Tables

**Table 1:** Generation-wise evolution of GSA approaches for microarray studies.

Approach	Methodology	Advantages	Limitations	Tools/Algorithms
**Over Representation Analysis** **(First generation microarrays GSA)**	Hypergeometric distribution/Fisher’s test Binomial distribution, Chi-square distribution, etc.	Easiness in execution. Assigns easily interpretable measure like p-values to the whole gene set.	Highly dependent on threshold/cutoff value, which is at user’s discretion and hard to determine. Test statistic independent of genes differential expression score. Uses only most significant genes based on hard threshold and discards others, lead to information loss. Assumes each gene contribute equally to phenotype/trait. Assumes each gene as independent and ignores the correlation or redundancy among genes in gene set. Assumes that each predefined gene set is independent of others, which is erroneous.	DAVID [41], AgriGO [32], Onto-Express [21], GenMAPP [42], GoMiner [43], FatiGO [44], GOstat [20], FuncAssociate [19], GOToolBox [45], GeneMerge [46], GOEAST [47], ClueGO [48], FunSpec [49], GARBAN [50], GO:TermFinder [22], WebGestalt [51], GOFFA [52], WEGO [53], GOTM [54], EASE, GSAQ [10], Pathview [55], Wholepathwayscope [56], ShinnyGO
**Enrichment Statistic Analysis** **(Second generation microarrays GSA)**	Wilcoxon signed rank test, Sum, Mean, or Median of gene-level statistic(s), Wilcoxon signed rank sum, Max-Mean Statistic	Do not require a threshold/ cutoff value for dividing gene space into selected and non-selected part. Considers dependence among genes in gene set. Test statistic is based on the differential GE score of genes in gene set.	Analyzes each gene set independently. Considers only the number of genes in a gene set (pathway) for performing GSA but ignores the additional information available from the bio-knowledge bases. Assumes the predefined gene sets mutually exclusive, but in biology, these gene sets are overlapping. Most ESA methods use differential GE to rank genes/compute test statistic but discard this information from further analysis.	GSEA [24], SAFE [39], GSA [8], Random set [57], sigPathway, Category, GlobalTest [26], PCOT2 [58], SAM-GS [59], LIMMA [60], Catmap [61], T-profiler [62], FunCluster [63], GeneTrail [64], Gazer [65], GSAQ [10], ANCOVA test, CAMERA [66], PAGE [37], GAGE [67], SGSE [68], GSNCA [69], GSA-SDR [70], GenePattern [71], plantGSEA [9], GSAR [29]
**Topology Analysis** **(Third generation microarrays GSA)**	**Graph/network theory**	Considers both genes relation /dependency with other genes as well as experimental condition changes. Considers the topology of the pathways/gene sets in modeling.	Dependent on the type of cell due to cell-specific GE profiles and condition being studied, which is rarely available. Not so popular as require more rarely available information and computationally intensive. Unable to consider interactions between gene sets (pathways). Heavily dependent on annotations.	PathwayExpress [72], ScorePAGE [73], SPIA [74], NetGSA [75], TopoGSA [76], CliPPER [77]

**Table 2:** Generation-wise evolution of GSA approaches for RNA-sequencing studies.

Approach	Methodology	Advantages	Limitations	Tools
**Over Representation Analysis** **(First generation RNA-seq GSA)**	Hypergeometric distribution, Fisher’s exact test	Simple to use. Assigns easily interpretable measure like *p-value* to the whole gene set. Less time consuming to interpret huge RNA-seq data.	Use hard threshold approach to select gene sets. Assumes each transcript as independent and ignores the correlation or gene-gene interaction. Mostly dependent on annotation bases, but RNA-seq transcripts are not well annotated.	GoSeq [80], iDEP [81]
**GS Enrichment Analysis** **(Second generation of RNA-seq GSA)**	Wilcoxon signed rank test, Max-Mean Statistic (with count normalization technique)	Do not require a threshold for dividing gene space into selected and non-selected part. Considers dependence among genes in gene set.	Use normalization technique to get microarray like data, hence, loss of the count nature of RNA-seq data Through data transformation, dispersion and other inherent nature of RNA-seq data are lost ES based tools/algorithms use differential score to prepare ranked transcript list but ignore this information for gene set testing. GSEA based tools like seqGSEA are computationally intensive, time consuming and and only offers the single gene set-level statistic. GSVA is not designed for gene set-based differential expression analysis between two phenotypically distinct sample groups. ES based GSA approaches do not consider the inherent zero inflation in the RNA-seq data.	AbsFilterGSEA [85], GSAAseqSP [86], seqGSEA [87], ssGSEA, EGSEA [88], GSVA [83], GSEPD [89], RNA-Enrich [90]

**Table 3:** Generation-wise evolution of GWAS GSA approaches for SNP data analysis.

Approach	Methodology	Advantages	Limitations	Tools/Algorithm
**Over Representation Analysis** **(First generation GWAS GSA)**	Hypergeometric distribution, Fisher’s exact test, Binomial test	Simple to use and easy to interpret Assigns statistically convincing measure like p-value for SNP set, which is biologically meaningful Computationally not so expensive	Hard threshold (arbitrary) divides the SNP list into selected and not selected SNP set. For instance, if threshold value for p-value is 0.05, means SNP with value 0.051 is not included in SNP list Uses only most significant SNP and discards others, lead to information loss Test statistic is independent of SNP data (based on only SNP count), and ignores the strength of association Considers each SNP independent and ignores the linkage disequilibrium Assumes each SNP contribute equally, which is not true as there are common and rare variants Dependent on pre-defined bio-knowledge base, which is mostly incomplete or unavailable	SNPtoGO [104], ALIGATOR [105], ATRP [106], MetaCore [107], PARIS [108], SET SCREEN test [109], SNP ratio test [99], GLOSSI, GeSBAP [98], INRICH [110], GeneSetDB [111], MAGENTA [112], KGG-HYST [113], PLINK [114], JAG [115], FORGE [116]
**Enrichment Statistic(s) Analysis** **(Second generation GWAS GSA)**	Wilcoxon signed rank test, Sum test, Weighted Sum test (Enrichment score like statistic)	Do not require hard threshold for dividing SNP list into selected and non-selected part Jointly consider multiple contributing factors in the same gene set, might complement the most-significant SNPs/genes approach Test statistic is computed from the SNP data considering linkage disequilibrium	Analyzes each gene set independently. Only considers data for selecting SNPs and after ignores the data from gene-set testing. Treat all genes in a gene set independently and do not account for the relationships between genes.	GSA-SNP [92], GSA-SNP2, GSEA-SNP [117], GSEA-P [118] GenGen [15], ICSNPathway [119], i-GSEA4GWAS [120], i-GSEA4GWAS2 [121]
**Topology Analysis** **(Third generation GWAS GSA)**	Graph/Network theory	Relationships between genes are used to assign different levels of “importance” to genes in the set Helps in integrate gene set membership information with interaction data from a separate source	Difficult to generalize True topology is dependent on the type of cell and experimental condition, which are rarely available Cannot model the dynamicity of the cellular system Heavily dependent on annotations, which is either missing or incomplete	dmGWAS [122], Ingenuity Pathway Analysis (IPA) [123], PINBPA [124], PathVisio [125], Cytoscape [126]
**Multivariate/Model/ Regression Analysis ** **(Fourth generation GWAS GSA)**	Linear regression Model, Ridge regression, Logistic regression, Linear models	Consider both SNP and gene set information simultaneously in same model Jointly consider linkage disequilibrium and gene-gene interaction in gene set for modeling Future behavior of the system can be predicted Dynamicity of the biological system can also be modeled and studied	Computationally intensive High dimensionality of genomic data raises serious concerns Ignores the non-linear interactions among biomolecules	LRpath [102], SPCA [100], SFPCA [101], MAGMA [127], GRASS, GeneralizedBerk-Jones statistic [103],

## Supplementary Materials

The following are available online at https://www.mdpi.com/1099-4300/22/4/427/s1, Document S1: Background methodologies of GSA approaches and tools for different generation, Figure S1: Operational procedures for gene set analysis followed in microarrays, RNA-seq, and GWAS data analysis, Figure S2: Analytical steps of GSA for microarray data analysis, Figure S3: Analytical steps of GSA for RNA-seq data analysis. Figure S4. Analytical steps of GSA for SNP (GWAS) data analysis. Table S1: List of available bio-knowledge bases used for Gene Set Analysis, Table S2: Nature and distribution of genomic datasets, Table S3: Available microarray datasets in NCBI, Table S4: Alternate annotation information for possible gene set analysis.
