# Supplementary material for: Fifteen Years of Gene Set Analysis for High-Throughput Genomic Data: A Review of Statistical Approaches and Future Challenges
Source: Entropy (Basel). 2020 Apr 10;22(4):427. doi: 10.3390/e22040427 (PMC7516904; doi:10.3390/e22040427)

Fifteen Years of Gene Set Analysis for High-Throughput Genomic Data: A Review of Statistical Approaches and Future Challenges - Supplementary information

Samarendra Das [1,2,5], Craig J. McClain [3,4,7,8,9] and Shesh N. Rai [2,4,5,6,7,*]

[1] Division of Statistical Genetics, ICAR-Indian Agricultural Statistics Research Institute, New Delhi 110012, India
[2] School of Interdisciplinary and Graduate Studies, University of Louisville, Louisville, KY 40292, USA
[3] Department of Medicine, University of Louisville, Louisville, KY 40202, USA
[4] Hepatobiology & Toxicology Center, University of Louisville, Louisville, KY 40202, USA
[5] Biostatistics and Bioinformatics Facility, JG Brown Cancer Center, University of Louisville, Louisville, KY 40202, USA
[6] Department of Bioinformatics and Biostatistics, University of Louisville, Louisville, KY 40202, USA
[7] Alcohol Research Center, University of Louisville, Louisville, KY 40202, USA
[8] Department of Pharmacology and Toxicology, University of Louisville, Louisville, KY 40202, USA
[9] Robley Rex Louisville VAMC, Louisville, KY 40206, USA

* **E-mail:** samarendra.das@louisville.edu, craig.mcclain@louisville.edu, shesh.rai@louisville.edu

Document S1. Background methodologies of GSA approaches and tools for different generations.

First generation (Over representation analysis)

Over Representation Analysis (ORA), also called functional enrichment analysis, is used to identify an over-represented pathway/GO category with a list of given/differentially expressed genes obtained (from Microarray or RNA-seq) by using traditional statistical tests such as t-test. Similarly, for SNP data, it starts by selecting SNPs and mapping the interesting SNPs to the corresponding genes. This initial selection process is based on whether a SNP is mapped to the pathway or whether the SNP is susceptible to the disease. Depending on the results, ORA builds a 2 × 2 contingency table to conduct a hypergeometric test. The underlying statistical tests/methodologies for each of the tools is given as below.

Table A. Background methodologies for first generation GSA.

Test/Methodology	Mathematical description	Assumptions	Implemented tools
Hypergeometric test	$p_i = 1 - \sum \frac{\binom{M}{n}\binom{N-M}{n-m}}{\binom{N}{n}}$ *N:* Total number of genes in gene space, *n:* total number of genes in the gene set, *M*: total number of genes in *i-th* pathway/GO category and *m:* number of (gene set) genes contained in *i-th* pathway/GO	Sampling without replacement from finite gene space	FunSpec, BINGO, CLENCH, FunSpec, GeneMerge, GFINDer, Onto-Express, GoMiner, FatiGO, GOTree Machine, GOToolBox, GeneMerge, ClueGO, THEA, STEM, Ontology Traverser, GOTM
Binomial test	$p_i = 1 - \sum_{i=0}^{m-1} \binom{k}{i}\left(\frac{M}{N}\right)^i \left(1-\frac{M}{N}\right)^{m-i}$	Dichotomous and nominal Independence	CLENCH, GFINDer, Onto-Express, THEA, L2L, GO TermFinder
Chi-square test	$\sum_{i \in P, i=I}^{\lvert P \rvert} (p_i - \bar{p})^2$	Independence Normality	CLENCH, Onto-Express, GOEAST, GOstat, NetAffx GO Mining Tool, GoSurfer

Fisher's exact test	$p = 1 - \frac{(a+b)!\,(a+c)!\,(b+d)!\,(c+d)!}{a!\,b!\,c!\,d!\,n!}$ a, b, c, d are cell entries of 2×2 table (a+b), (c+d), (a+c), (b+d) are 2×2 table rows and column sums.	Sampling without replacement from finite gene space	DAVID, eGOn, EASEonline, eGOn, FatiGO, FuncAssociate, GFINDer, GOseq, EVA, SNPtoGO, GESBAP, ALIGATOR

Second generation (Enrichment scoring statistic(s)):

The second-generation methods use a variation of a general framework, but have a common executional pattern, consists of the following steps: (i) a gene-level statistic is computed using the molecular measurements from an experiment; (ii) computation of gene set level statistic; (iii) Evaluation of statistical significance of the computed statistic. The underlying statistical tests/methodologies used in second generation GSA tools are given as below.

Table B. Background methodologies for second generation GSA.

**Test**	**Mathematical description**	**Applicability**	**Tools**	**Availability**
Wilcoxon signed rank test	Rank sum statistic: $\sum_{i\in P, i=I}^{\lvert P\rvert} rank_{P+Q}(p_i)$	Microarrays SNP, RNA-seq	sigPathway, SAFE	Web, R package
Weighted Kolmogorov-Smirnov	Test statistic: Maximum deviation (located at position i) between $\sum_{\substack{j\le i \\ j\in P}} \frac{r_j(P+Q)}{\sum_{k\in P} r_k(P+Q)}, \sum_{j\le i} \frac{1}{\lvert Q\rvert}$	Microarrays RNA-seq, SNP	SAFE, GSEA, seqGSEA, GSEA-SNP, i-GSEA4GWAS, GSEPD	R package
Mean test	$\frac{1}{\lvert P\rvert} \sum_{i\in P, i=I}^{\lvert P\rvert} p_i$	Microarrays	GSEA	R package
Median test	$\begin{cases} y_{\frac{n}{2}}(P) \ \ if\ n\ is\ odd \\ \frac{y_{\frac{n}{2}}(P) + y_{1+\frac{n}{2}}(P)}{2} \ \ if\ n\ is\ even \end{cases}$	Microarrays	GSEA	R package
Max-mean statistic	$S_{max} = max\left\{ \left\lvert \frac{\sum_{i=1}^{m} I(t_i > 0)t_j}{m} \right\rvert, \left\lvert \frac{\sum_{i=1}^{m} I(t_i < 0)t_j}{m} \right\rvert \right\}$	Microarrays, SNP	GSA	R package
Q-statistic	$Q = \frac{1}{m}\sum_{i=1}^{m} \frac{1}{\mu_2}[X_i(Y-\mu)]^2$	Microarrays	Global Test	
Hotelling's **T2**	Multivariate T-statistic	Microarrays	PCOT2	R package
Z-score	$Z = \frac{1}{\delta}(\mu - \mu_c)\sqrt{m}$ where, μ, δ are the mean and standard deviation of fold changes calculated for all genes and μ_c is the mean of fold changes for genes in G	Microarrays, SNP	PAGE, dmGWAS	
t-statistic	$z = \frac{1}{\sqrt{m}}\sum_{i=1}^{m} t_i$	Microarrays	CATEGORY	

Two sample t-test	$t = \frac{m - M}{\sqrt{\frac{s^2}{n} + \frac{S^2}{n}}}$	Microarrays	GAGE	R package
Non-parametric test statistic		RNA-seq	GSVA	

Third generation (Topology based):

Topology/Graph theory-based methods are similar to the second-generation methods as they perform the same steps as that of second generation methods. However, they only use pathway topology/gene set network information to compute gene-level statistics. The methodology used in third generation of GSA tools are given as:

Table C. Background methodologies for third generation GSA.

Test/Methodology	Mathematical description	Applicability	Tools	Availability
Graph (topology) theoretic approaches	Directed Acyclic Graph	Microarrays, SNP	dmGWA, Ingenuity Pathway Analysis (IPA), PINBPA, PathVisio, Cytoscape PathwayExpress, ScorePAGE, SPIA, NetGSA, TopoGSA, CliPPER	R package, Web

Fourth generation (Multivariate/Model based):

The second and third generations GSA tools take test statistic(s) or *p-values* associated with genes as input, while ignores the original nature (*i.e.* discrete, continuous, categorical) of genomics data. Thus, fourth generation of GSA approaches are being developed by providing original data as input. The underlying tests/methodology used in such tools are given as below.

Table D. Background methodologies for fourth generation GSA.

Test/Methodology	Mathematical description	Applicability	Tools	Availability
Linear model	$y_{gi} = \beta_{g0} + \sum_{j=1}^{p} X_{ij}\beta_{gj} + \varepsilon_{gi}$	Microarrays, SNP	GSEAlm, MAGMA	R package
Logistic Regression	$\frac{p}{1-p} = \beta_{g0} + \sum_{j=1}^{p} X_{ij}\beta_{gj} + \varepsilon_{gi}$	Microarrays, SNP	LRpath, Logistic kernel machine regression Generalized Berk-Jones statistic[37],	R package
Regularized regression	$y_{gi} = \beta_{g0} + \sum_{j=1}^{p} X_{ij}\beta_{gj} + \varepsilon_{gi}$	Microarray, SNP	GRASS, gerr	R package

	With regularization (1) Lasso (L1 regularization) (2) Ridge, (L2 regularization) and (3) the elastic net (hybrid of L1 and L2 regularization)			
Principal component based approaches	Principal Component Analysis (PCA), Smooth PCA, Smooth Functional PCA	Microarray,SNP	A two-stage approach SNP SPCA SNP, SPCA, SFPCA,	R package
Bayesian model	Bayes theorem	Microarrays	GOing Bayesian	R package

Table S1. List of available bio-knowledge bases used for Gene Set Analysis.

Name	Description	URL	Ref.
**BioCarta**	Users input research data to construct the knowledge base	http://www.biocarta.com	
**Gene Ontology (GO)**	Large hierarchy of terms representing biological concept	http://geneontology.org/	[1,2]
**KEGG**	Provides higher-order (genomic and pathway annotations) information from input of molecular data for various organisms	http://www.genome.jp/kegg/	[3]
**MetaCore**	Extensive pathways derived from publications. Allows users to modify pathway elements for illustration purpose	http://thomsonreuters.com/metacore/	[4]
**MetaCyc**	Contains metabolic and enzymatic pathways from various organisms experimentally validated in literature	http://metacyc.org/	[5]
**MSigDB**	Contains a collection of annotated gene sets for use with their GSEA software. The collection includes various gene sets defined by biological functions, GO, KEGG, positions, sequence regulation information *etc*.	http://www.broadinstitute.org/gsea/msigdb/	[6]
**Pathway Interaction Database (PID)**	A highly structured, curated collection of information about known biomolecular interactions and key cellular processes assembled into signaling pathways	http://pid.nci.nih.gov/	[7]
**REACTOME**	Provides a platform for annotating and visualizing data from major databases such as NCBI Gene, Ensembl and UniProt databases, UCSC & HapMap Genome Browsers, KEGG Compound and ChEBI small molecule databases, PubMed and GO	http://www.reactome.org/	[8]
BIOPATH	Database of biochemical pathways that provides access to metabolic transformations and cellular regulations	https://www.mn-am.com/databases/biopath	[9]
MPW	The Metabolic Pathways Database	www.biobase.com/emphome.html/homepage	[10]
EMP	An encoding of the contents of over 10 000 original publications on the topics of enzymology and metabolism. An extraction of over 1800 pictorial representations of metabolic pathways. This collection plays an important role in the interpretation of genetic sequence data, as well as offering a meaningful framework for the integration of many other forms of biological data.	http://emp.mcs.anl.gov	[11]
**CSNDB**	Provides all biological properties of cellular signal transduction pathways, including biological pathways that transfer cellular signals and molecular attributes characterized by sequences, structures and functions.	https://omictools.com/csndb-tool	[12]
**SPAD**	Protein signaling cascades with pathway diagrams for a limited number of extracellular signaling pathways in three broad areas: growth factors, cytokines and hormones. Each component of a pathway is hyperlinked to a page containing further details.	http://www.grt/spad	

**TRANSPATH**	Offers information about the intracellular signaling pathways. It allows the user to see details of the signal flow from the cell surface into the nucleus, focusing on mammals such as humans, mice and rats.	http://www.biobase.de/pages/products/databases.html	[13]
**BBID**	Database of biological images. This contains images of all sorts including pathways, structures, gene famies and cellular structures. Users can search keyword or browse by represented genes or the entire list of available keywords.	http://bbid.grc.nia.nih.gov/	[14]
**HPRD**	Database of curated proteomic information pertaining to human proteins.	http://www.hprd.org	[15]
**STKE**	Useful for researchers interested in exploring canonical pathways, the scope of the networks and complexity of the regulatory events involved in cellular signaling pathways. AAAS decided to focus efforts in other areas of scientific communication and is not redeveloping or updating the data in the Database or the data entry software.	http://dictybase.org/STKE.html	
**BRITE**	Collection of hierarchical files capturing functional hierarchies of various biological pathways. In contrast to KEGG pathway, which is limited to molecular interactions and reactions, BRITE incorporates many different types of relationships including: genes and proteins, compounds and Reactions, drugs, diseases and organisms and cells	http://www.genome.ad.jp/brite/	
**TRANSFAC**	Manually curated database of eukaryotic transcription factors, their genomic binding sites and DNA binding profiles. This can be used to predict potential transcriptional regulation pathway.	http://transfac.gbf-braunschweig.de	[16]
**CST**	Contains interactive signaling pathway diagrams, research overviews, relevant antibody products, publications, etc. Protein nodes in each interactive pathway diagram are linked to specific antibody product information or, optionally, to protein-specific listings in the database of post-translational modifications.	https://www.cellsignal.com/contents/science/cst-pathways/science-pathways	
**Database of Interacting Proteins (DIP)**	Catalogs the experimentally determined interactions between proteins. It combines information from a variety of sources to create a single, consistent set of protein-protein interactions. The data stored within the DIP database were curated, both, manually by expert curators and automatically using computational approaches that utilize the knowledge about the protein-protein interaction networks extracted from the most reliable, core subset of the DIP data.	https://dip.doe-mbi.ucla.edu/dip/Main.cgi	[17]
**Gramene**	Open source, curated resource for plant comparative genomics and pathway analysis designed to support researchers working in plant genomics, breeding, evolutionary biology, system biology, and metabolic engineering. It consists of genomic information visualizing and analyzing data for 44 plant including curated rice pathways and orthology-based pathway projections for 66 plant species including various crops.	www.gramene.org	[18]

**PANTHER**	Classification System is designed to classify proteins (and their genes) in order to facilitate high-throughput analysis.	http://www.pantherdb.org/about.jsp	[19]
**INOH**	Highly structured, manually curated database of signal transduction pathways including Mammalia, *Xenopus laevis*, *Drosophila melanogaster*, *Caenorhabditis elegans* and canonical.	http://www.inoh.org/	[20]
**NetPath**	A resource of curated human signaling pathways. Also provides detailed maps of a number of immune signaling pathways. Act as a consolidated resource for human signaling pathways that should enable systems biology approaches.		[21]
**GOLD.db**	Provides biological pathways with image maps and visual pathway information for lipid metabolism and obesity-related research. This database provides also the possibility to map gene expression data individually to each pathway. Gene expression at different experimental conditions can be viewed sequentially in context of the pathway.	http://gold.tugraz.at	[22]
**PATIKA**	Patika is composed of a server-side, scalable, object-oriented database and client-side editors to provide an integrated, multi-user environment for visualizing and manipulating network of cellular events. This tool features automated pathway layout, functional computation support, advanced querying and a user-friendly graphical interface.	patika@cs.bilkent.edu.tr	[23]
**pSTIING**	Knowledgebase featuring 65 228 distinct molecular associations (comprising protein–protein, protein–lipid, protein–small molecule interactions and transcriptional regulatory associations), ligand–receptor–cell type information and signal transduction modules.	http://pstiing.licr.org	[24]
**TRMP**	Information about non-target proteins and natural small molecules involved in these pathways also provides useful hint for searching new therapeutic targets and facilitate the understanding of how therapeutic targets interact with other molecules in performing specific tasks. The TRMPs database is designed to provide information about such multiple pathways along with related therapeutic targets, corresponding drugs/ligands, targeted disease conditions, constituent individual pathways, structural and functional information about each protein in the pathways.	http://bidd.nus.edu.sg/group/trmp/trmp.asp	[25]
**WikiPathways**	Open, collaborative platform dedicated to the curation of biological pathways. Presents a new model for pathway databases that enhances and complements ongoing efforts, such as KEGG, Reactome and Pathway Commons. A custom graphical pathway editing tool and integrated databases covering major gene, protein, and small-molecule systems are also available. The familiar web-based format of WikiPathways greatly reduces the barrier to participate in pathway curation.	https://www.wikipathways.org	[26]

**The Cancer Cell Map**	Ten human cancer-related signaling pathways	https://cancer.cellmap.org/cellmap	[27]
**HPD**	Human Pathway Database (HPD) by integrating heterogeneous human pathway data that are either curated at the NCI Pathway Interaction Database (PID), Reactome, BioCarta, KEGG or indexed from the Protein Lounge Web sites.	http://bio.informatics.iupui.edu/HPD	[28]

References

[1] Gene Ontology Consortium 2004 The Gene Ontology (GO) database and informatics resource *Nucleic Acids Res.*

[2] Ashburner M, Ball C A, Blake J A, Botstein D, Butler H, Cherry J M, Davis A P, Dolinski K, Dwight S S, Eppig J T, Harris M A, Hill D P, Issel-Tarver L, Kasarskis A, Lewis S, Matese J C, Richardson J E, Ringwald M, Rubin G M and Sherlock G 2000 Gene Ontology: tool for the unification of biology *Nat. Genet.* **25** 25–9

[3] Kanehisa M 2004 The KEGG resource for deciphering the genome *Nucleic Acids Res.* **32** 277D – 280

[4] Schuierer S, Tranchevent L C, Dengler U and Moreau Y 2010 Large-scale benchmark of Endeavour using MetaCore maps *Bioinformatics*

[5] Caspi R 2005 MetaCyc: a multiorganism database of metabolic pathways and enzymes *Nucleic Acids Res.*

[6] Liberzon A, Subramanian A, Pinchback R, Thorvaldsdottir H, Tamayo P and Mesirov J P 2011 Molecular signatures database (MSigDB) 3.0 *Bioinformatics* **27** 1739–40

[7] Schaefer C F, Anthony K, Krupa S, Buchoff J, Day M, Hannay T and Buetow K H 2009 PID: The pathway interaction database *Nucleic Acids Res.*

[8] Croft D, Mundo A F, Haw R, Milacic M, Weiser J, Wu G, Caudy M, Garapati P, Gillespie M, Kamdar M R, Jassal B, Jupe S, Matthews L, May B, Palatnik S, Rothfels K, Shamovsky V, Song H, Williams M, Birney E, Hermjakob H, Stein L and D'Eustachio P 2014 The Reactome pathway knowledgebase *Nucleic Acids Res.*

[9] Brandenburg F J, Forster M, Pick A, Raitner M and Schreiber F 2004 BioPath — Exploration and Visualization of Biochemical Pathways pp 215–35

[10] Selkov E 1998 MPW: the Metabolic Pathways Database *Nucleic Acids Res.* **26** 43–5

[11] Selkov E, Basmanova S, Gaasterland T, Goryanin I, Gretchkin Y, Maltsev N, Nenashev V, Overbeek R, Panyushkina E, Pronevitch L, Selkov E and Yunus L 1996 The metabolic pathway collection from EMP: The enzymes and metabolic pathways database *Nucleic Acids Res.*

[12] Takai-Igarashi T and Kaminuma T 1999 A pathway finding system for the cell signaling networks database. *In Silico Biol.* **1** 129–46

[13] Krull M, Voss N, Choi C, Pistor S, Potapov A and Wingender E 2003 TRANSPATH®: An integrated database on signal transduction and a tool for array analysis *Nucleic Acids Res.*

[14] Becker K G, White S L, Muller J and Engel J 2000 BBID: the biological biochemical image database *Bioinformatics* **16** 745–6

[15] Keshava Prasad T S, Goel R, Kandasamy K, Keerthikumar S, Kumar S, Mathivanan S, Telikicherla D, Raju R, Shafreen B, Venugopal A, Balakrishnan L, Marimuthu A, Banerjee S, Somanathan D S, Sebastian A, Rani S, Ray S, Harrys Kishore C J, Kanth S, Ahmed M, Kashyap M K, Mohmood R, Ramachandra Y L, Krishna V, Rahiman B A, Mohan S, Ranganathan P, Ramabadran S, Chaerkady R and Pandey A 2009 Human Protein Reference Database--2009 update *Nucleic Acids Res.* **37** D767–72

[16] Matys V 2006 TRANSFAC(R) and its module TRANSCompel(R): transcriptional gene regulation in eukaryotes *Nucleic Acids Res.*

[17] Xenarios I 2000 DIP: the Database of Interacting Proteins *Nucleic Acids Res.*

[18] Ware D 2002 Gramene: a resource for comparative grass genomics *Nucleic Acids Res.*

[19] Thomas P D, Campbell M J, Kejariwal A, Mi H, Karlak B, Daverman R, Diemer K, Muruganujan A and Narechania A 2003 PANTHER: a library of protein families and subfamilies indexed by function. *Genome Res.*

[20] Yamamoto S, Sakai N, Nakamura H, Fukagawa H, Fukuda K and Takagi T 2011 INOH: Ontology-based highly structured database of signal transduction pathways *Database*

[21] Kandasamy K, Mohan S, Raju R, Keerthikumar S, Kumar G S S, Venugopal A K, Telikicherla D, Navarro D J, Mathivanan S, Pecquet C, Gollapudi S K, Tattikota S G, Mohan S, Padhukasahasram H, Subbannayya Y, Goel R, Jacob H K C, Zhong J, Sekhar R, Nanjappa V, Balakrishnan L, Subbaiah R, Ramachandra Y L, Rahiman A, Keshava Prasad T S, Lin J-X, Houtman J C D, Desiderio S, Renauld J-C, Constantinescu S, Ohara O, Hirano T, Kubo M, Singh S, Khatri P, Draghici S, Bader G D, Sander C, Leonard W J and Pandey A 2010 NetPath: a public resource of curated signal transduction pathways *Genome Biol.* **11** R3

[22] Hackl H, Maurer M, Mlecnik B, Hartler J, Stocker G, Miranda-Saavedra D and Trajanoski Z 2004 GOLD.db: Genomics of lipid-associated disorders database *BMC Genomics*

[23] Demir E, Babur O, Dogrusoz U, Gursoy A, Nisanci G, Cetin-Atalay R and Ozturk M 2002 PATIKA: An integrated visual environment for collaborative construction and analysis of cellular pathways *Bioinformatics*

[24] Ng A 2006 pSTIING: a "systems" approach towards integrating signalling pathways, interaction and transcriptional regulatory networks in inflammation and cancer *Nucleic Acids Res.* **34** D527–34

[25] Zheng C J, Zhou H, Xie B, Han L Y, Yap C W and Chen Y Z 2004 TRMP: A database of therapeutically relevant multiple pathways *Bioinformatics*

[26] Pico A R, Kelder T, van Iersel M P, Hanspers K, Conklin B R and Evelo C 2008 WikiPathways: Pathway Editing for the People *PLoS Biol.*

[27] Tsherniak A, Vazquez F, Montgomery P G, Weir B A, Kryukov G, Cowley G S, Gill S, Harrington W F, Pantel S, Krill-Burger J M, Meyers R M, Ali L, Goodale A, Lee Y, Jiang G, Hsiao J, Gerath W F J, Howell S, Merkel E, Ghandi M, Garraway L A, Root D E, Golub T R, Boehm J S and Hahn W C 2017 Defining a Cancer Dependency Map *Cell*

[28] Chowbina S R, Wu X, Zhang F, Li P M, Pandey R, Kasamsetty H N and Chen J Y 2009 HPD: an online integrated human pathway database enabling systems biology studies *BMC Bioinformatics* **10** S5

Table S2. Nature and distribution of genomic datasets.

**Genomic Study**	**Nature of data**	**Prob. distribution**
Microarrays	Continuous	Gaussian
RNA-Seq	Discrete (Count)	Negative Binomial
GWAS	Binary	Binomial

Table S3. Available Microarray datasets in NCBI.

Attributes	Public	Unreleased	Total
Series	112,050	13,326	125,376
Platforms	19,664	229	19,893
Samples	3,004,081	402,137	3,406,218

(Data taken up to May 15, 2019)

Table S4. Alternate annotation information for possible gene set analysis.

Annotation	Possible hypothesis(s)	Description
Chromosomal location	**Self-contained *H_0***: No genes in gene set are overlapped with a particular chromosomal location(s) differentially expressed.<br><br>**Competitive *H_0***: Genes in gene set are at most as often overlapped with a particular chromosomal location(s) as the genes not in gene set	Here, a gene set (as the collection of genes) can be tested for their association with the chromosomal locations (e.g. on chromosome 1). Therefore, proper statistical approach and tools need to be developed to analyze gene sets with respect to annotation information like chromosomal locations.
Differential expression	**Self-contained *H_0***: No genes in gene set are differentially expressed.<br><br>**Competitive *H_0***: Genes in gene set are at most as often overrepresented with the differentially expressed genes as the genes not in gene set	In usual differential expression analysis, differentially expressed gene list and differential expression score is computed for each gene. Further, statistical methodology can be developed to test whether the gene set is overrepresented in this list.
Quantitative Trait Loci (QTL)	**Self-contained *H_0***: No genes in the gene set are over-lapped with the QTL regions.<br><br>**Competitive *H_0***: Genes in gene set are at most as often overlapped with the QTL regions as the genes not in gene set	QTLs are segment of genomic regions either containing or linked to genes that correlates with variation in a phenotype.<br>Performing analysis of gene sets based on trait specific QTLs through a computational approach instead of traditional GO or pathways information will be very helpful in unraveling genotype-phenotype relationships.
Exon content	**Self-contained *H_0***: Genes in the gene set are enriched with equal exon content.<br><br>**Competitive *H_0***: Genes in gene set are at most as often enriched with equal exon content as the genes not in gene set	Another set of statistical tests can be designed to test the gene sets with respect to exon count. For instance, a null hypothesis can be such that genes in the gene sets have higher proportions of exon counts as compared to that of outside the gene sets.
Biological process (e.g. cell cycle)	**Self-contained *H_0***: No genes in the gene set are represented with a biological process (e.g. cell cycle).<br><br>**Competitive *H_0***: Genes in gene set are at most as often overrepresented with the biological process (e.g. cell cycle) as the genes not in gene set	Gene sets can be tested for their association with a biological process (e.g. cell cycle). Therefore, proper statistical approach and tools need to be developed to analyze gene sets with respect to cell cycle like information.
Condition/ Disease type/ Cell type	**Self-contained *H_0***: No genes in the gene set are associated with a particular disease type.	A gene set (as the collection of genes) can be tested for their association with the disease type (*e.g.* breast

	**Competitive** ***H_0***: Genes in gene set are at most as often associated with a particular disease type as the genes not in gene set	cancer or lung cancer). Therefore, proper statistical approach and tools need to be developed to analyze gene sets with respect to disease information.

H_0: Null hypothesis

Figure S1. Standard operation procedures for gene set analysis followed in microarrays, RNA-seq and GWAS.

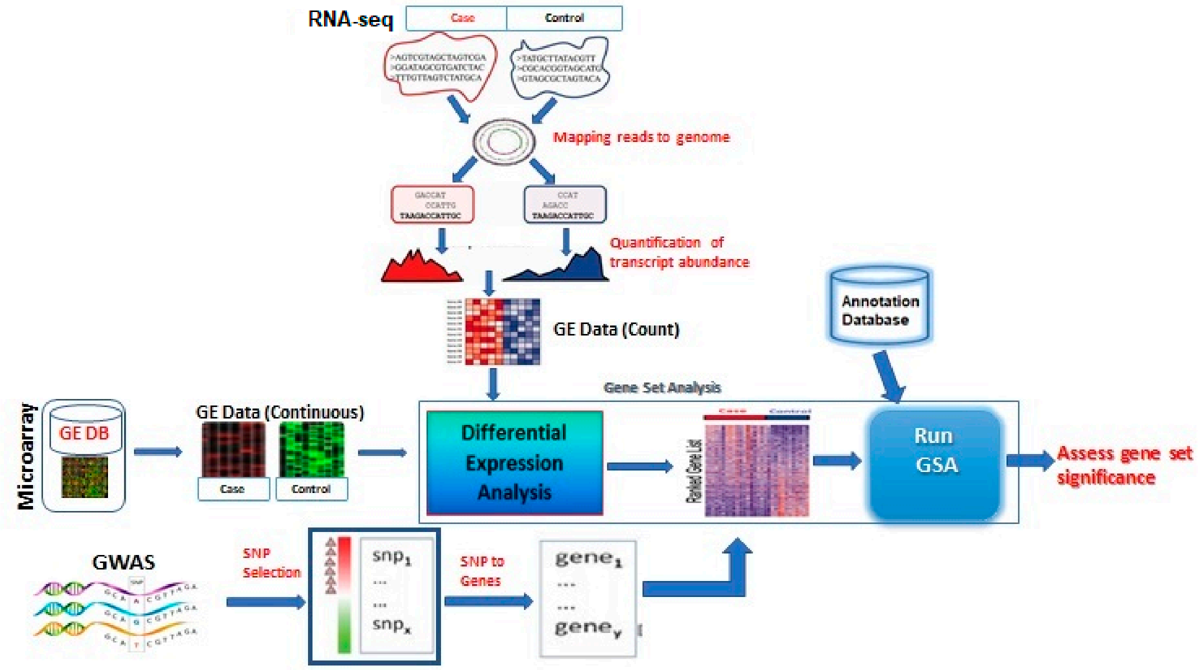

Figure S2. Analytical steps of GSA for microarray data analysis.

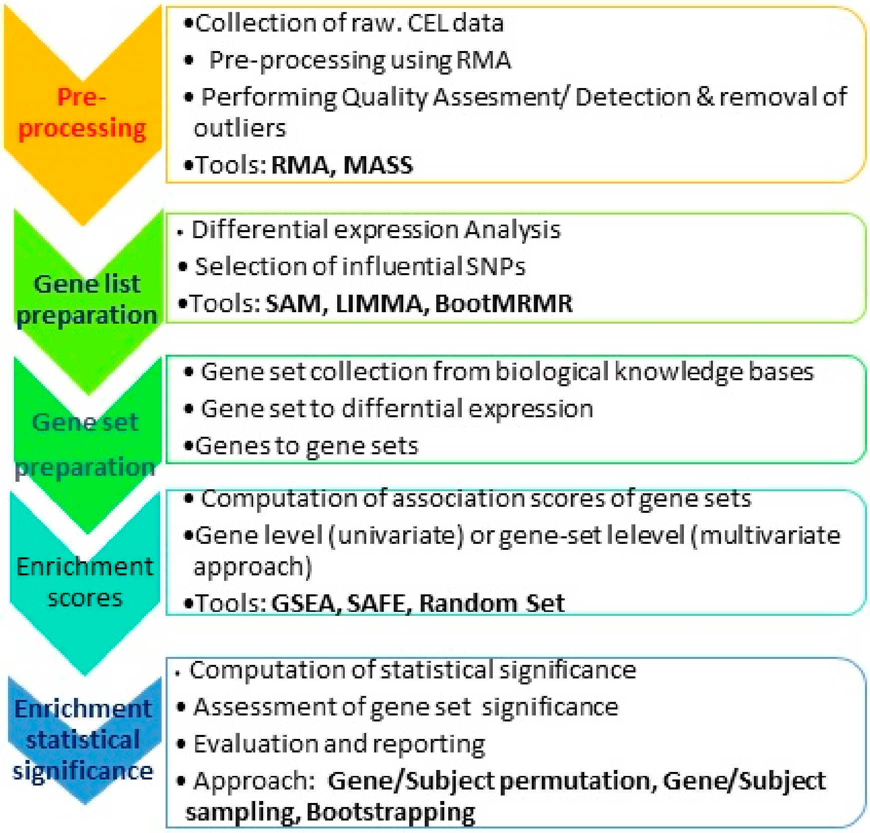
Pre-processing
•Collection of raw. CEL data
• Pre-processing using RMA
• Performing Quality Assesment/ Detection & removal of outliers
•Tools: RMA, MASS
Gene list preparation
• Differential expression Analysis
• Selection of influential SNPs
•Tools: SAM, LIMMA, BootMRMR
Gene set preparation
• Gene set collection from biological knowledge bases
• Gene set to differntial expression
•Genes to gene sets
Enrichment scores
• Computation of association scores of gene sets
•Gene level (univariate) or gene-set lelevel (multivariate approach)
•Tools: GSEA, SAFE, Random Set
Enrichment statistical significance
• Computation of statistical significance
• Assessment of gene set significance
• Evaluation and reporting
• Approach: Gene/Subject permutation, Gene/Subject sampling, Bootstrapping

Figure S3. Analytical steps of GSA for RNA-seq data analysis.

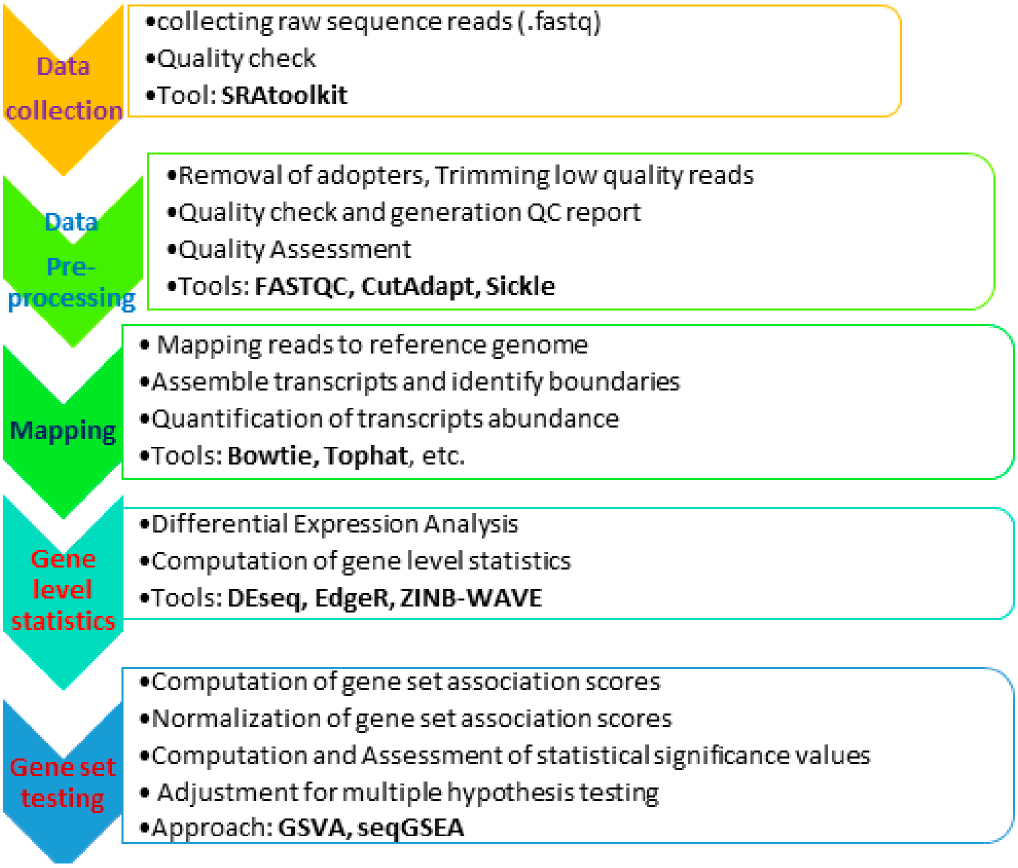

Figure S4. Analytical steps of GSA for SNP (GWAS) data analysis.

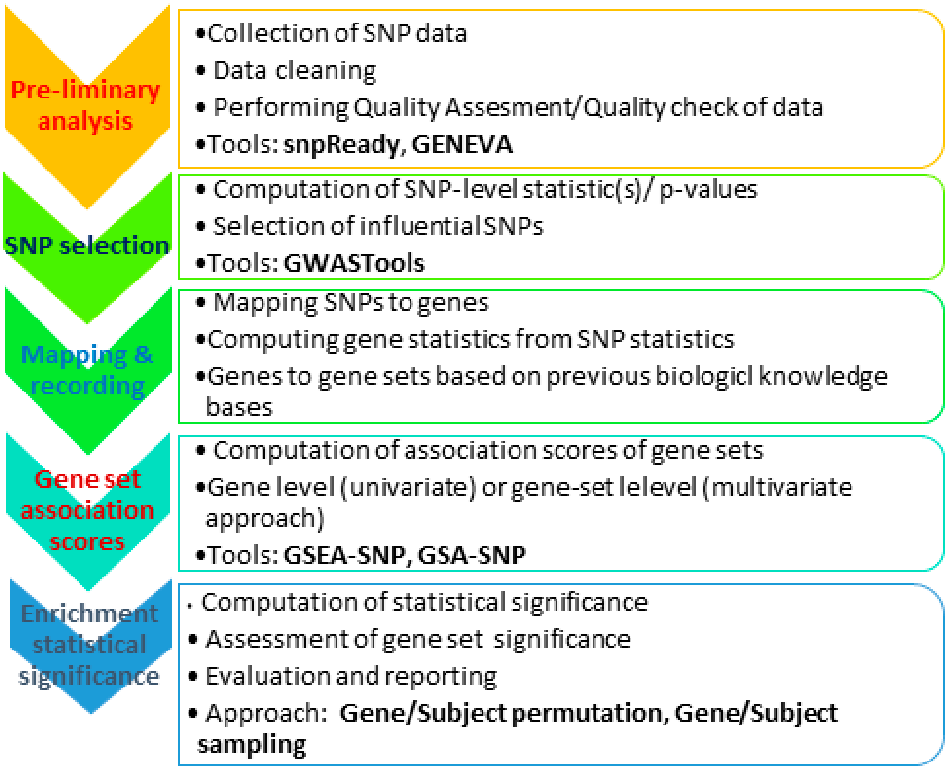

Supplement: Supplementary file 1 [file entropy-22-00427-s001.pdf]
